# The Value of Some Lactic Acid Ferments

**Published:** 1909-03

**Authors:** Walker Hall, W. A. Smith


### THE VALUE OF SOME LACTIC ACID FERMENTS.

BY

Drs. Walker Hall and W. A. Smith.

They showed that there is a great variability in the activity and power of the individual tablets sold commercially, and that a large number of the preparations are inert. Speaking generally, it appeared that when raw, pasteurised or partly-sterilised milk is kept at blood-heat for ten to twenty hours the same amount of acidity is developed as when tablets of lactic acid ferments are added. The organisms contained in the tablets seem to require a considerable time to overcome the normal and abnormal organisms of milk, and the development of their powers is so slow, that for the purposes of lactic acid therapy they are of little avail. Few of them grow well in artificial gastric juice, and when mixed with an emulsion of fæcal anaërobic and aërobic bacteria only two out of ten types were alive after five days' incubation. It was pointed out that more satisfactory results may be obtained with living cultures, but that it is necessary first to subject the organism to aërobic and anaërobic special enrichment media; by these means cultures had been obtained which appeared in the fæces within twenty-four hours after administration by the mouth. Before lactic acid therapy is undertaken it was found advisable to ascertain that the fæcal anærobes are in excess; otherwise the administration of lactic acid bacilli may be harmful rather than curative. The authors of the paper found that 5 c.c. of a twenty-four hours' growth of a strong culture in their enrichment medium given in the early morning and late evening sufficed to guarantee the presence of the lactic acid bacilli in the intestine for a week. This was made easy of demonstration by a simple method of isolation, which they described. This weekly dosage, it was claimed, would make the treatment a definite one, would save a considerable amount of trouble as compared with the daily souring of milk, and would reduce the dangers associated with the sterilisation and incubation of milk. In addition, the cost of treatment would be diminished, as it would be unnecessary to prescribe the continuous taking of the ferment tablets. They also stated that if "soured" milk was desirable from the standpoint of digestion, the enriched cultures they showed produced in a shorter time a more finely divided curd than that which resulted from the addition of the dry tablets.

